# RAS-associated Autoimmune Leukoproliferative disease (RALD) manifested with early-onset SLE-like syndrome: a case series of RALD in Chinese children

**DOI:** 10.1186/s12969-019-0346-1

**Published:** 2019-08-14

**Authors:** Wei Wang, Yu Zhou, Linqing Zhong, Lin Wang, Xiaoyan Tang, Mingsheng Ma, Ji Li, Hongmei Song

**Affiliations:** 1 Department of Pediatrics, Peking Union Medical College Hospital, Chinese Academy of Medical Sciences, Beijing, China; 2 School of Medicine, Tsinghua University, Beijing, China

**Keywords:** NRAS, RALD, Somatic mutation, SLE

## Abstract

**Background:**

Primary immunodeficiency diseases (PIDs) patients may show systemic lupus erythematosus (SLE)-like autoimmunity disorders, such as cytopenias, as well as polyarthritis, which leads to concerns of misdiagnosis. We diagnosed three RALD cases between 2015 and 2018, who were suspected as SLE and summarized clinical characteristics.

**Methods:**

We collected and analyzed the clinical data of the 3 cases. DNA was extracted from the patients’ and their parents’ peripheral blood as well as oral mucosa cells, hair follicles, and nails. Genes were detected with the application of gene trapping high-throughput sequencing using PIDs panel and suspicious gene or mutation was further verified by Sanger sequencing.

**Results:**

1. Clinical features: On the one hand, the patients presented with severe thrombocytopenia, facial erythema, arthritis, positive autoantibodies and other manifestations, supporting the diagnosis of SLE. On the other hand, symptoms including early onset ages, recurrent infections, lymphadenopathy, hepatosplenomegaly, monocytosis and hypergammaglobulinemia, were common observed in PIDs.

2. Gene analysis: NRAS mutations (c.38G > A, p.G13D or c.37G > T, p.G13C) were found in the blood of the patients. Besides, the same set of mutations was detected in buccal mucosa of patient 1 and nails of patient 3 while the frequency was much lower. However, no mutation was found in other tissues or in their parents’ blood. Consequently, they were NRAS somatic mutated RALD.

**Conclusions:**

For those early-onset SLE-like patients with predominant hematologic disorders, monocytosis, recurrent infectious history, accompanied with hepatosplenomegaly and lymphadenopathy, a genetic screening of PIDs might be required.

## Background

Systemic lupus erythematosus (SLE) is a chronic autoimmunity disease, which exhibits a variety of clinical manifestations. Generally, patients with SLE usually present with multiple cutaneous manifestations like butterfly rash and discoid rash, nonerosive arthritis, serositis and disorders of almost all systems especially renal and neurologic system [1]. Primary immunodeficiency diseases (PIDs) are heterogeneous diseases consisting of more than 300 single-gene inherited disorders, whose symptoms are also diverse. The major clinical features of PIDs include infection, malignancy, allergy, auto-immunity, and auto-inflammation [2]. Some PIDs patients mainly show autoimmune phenotypes, including autoimmune cytopenias, polyarthritis, and positive autoantibodies, especially for those with autoimmune lymphoproliferative syndrome (ALPS) and type 1 interferonopathies in the diseases of immune dysregulation. And thus those patients might be misdiagnosed as SLE [3].

RAS-associated leukoproliferative disease (RALD), is a newly defined non-malignant lymphoproliferation disease which was initially classified into a subset of autoimmune lymphoproliferative syndrome (ALPS) [4]. Here we reported 3 patients who were initially suspected to be SLE with hematologic disorder but were finally diagnosed as RALD by gene trapping high-throughput sequencing detection.

## Methods

### Patients

We collected and analyzed the clinical characteristics of 3 cases. All patients were misdiagnosed as SLE in local hospital because of several SLE-like manifestations such as autoimmune thrombocytopenia, polyarthritis and positive autoantibodies. However, several uncommon manifestations in SLE were noticed, such as hepatosplenomegaly, lymphadenopathy, recurrent infections. Besides, taking the age of onset and the gender of those patients into account, all above indicated the possibility of PIDs and thus those patients received gene trapping high-throughput sequencing. This study was approved by the Institutional Review Board of Peking Union Medical College Hospital (PUMCH) and performed according to the Declaration of Helsinki. Informed consent was obtained from all participants.

### Gene trapping high-throughput sequencing

Peripheral blood DNA samples from 3 cases and their parents were extracted. For some PIDs have SLE-like symptoms, we detected 237 PIDs related genes from the patients by gene trapping high-throughput sequencing with the application of PIDs panel. Suspected genes were chosen according to the following criteria: Variations types were not synonymous mutations; MAF < 0.01; at least 1 variation in AD genetic pattern genes, 1 homozygous or 2 heterozygous variations in AR genetic pattern genes. If any suspected genes or mutations were found, we would verify the mutation locus of the patient and their parents by Sanger sequencing. Then we would search the mutations in HGMD and SNP database and speculate the pathogenicity of the mutations by SIFT and Polyphen.

### Somatic mutation detection by sanger sequencing

For suspected pathogenetic mutations found in NRAS, both germline and somatic mutation of NRAS may cause disease, we detected those mutations in other tissues to identify if those suspicious mutations were somatic mutations. DNA was extracted from the patients’ oral mucosa cells, hair follicles, and nails. PCR was performed using the protocol listed below: 95 °C for 5 min; followed by 35 PCR cycles (95 °C for 30 s, 59 °C for 30 s, 72 °C for 45 s; then 72 °C for 7 min). The primer-pairs sequences are: NRAS-E1-F: 5′-CCAGAAGTGTGAGGCCGATA-3′, NRAS-E1-R: 5′-TCGCTACTATGGCCTGTGTT-3′. PCR products were purified and Sanger sequencing tests were performed at last.

## Results

**Fig. 1:**
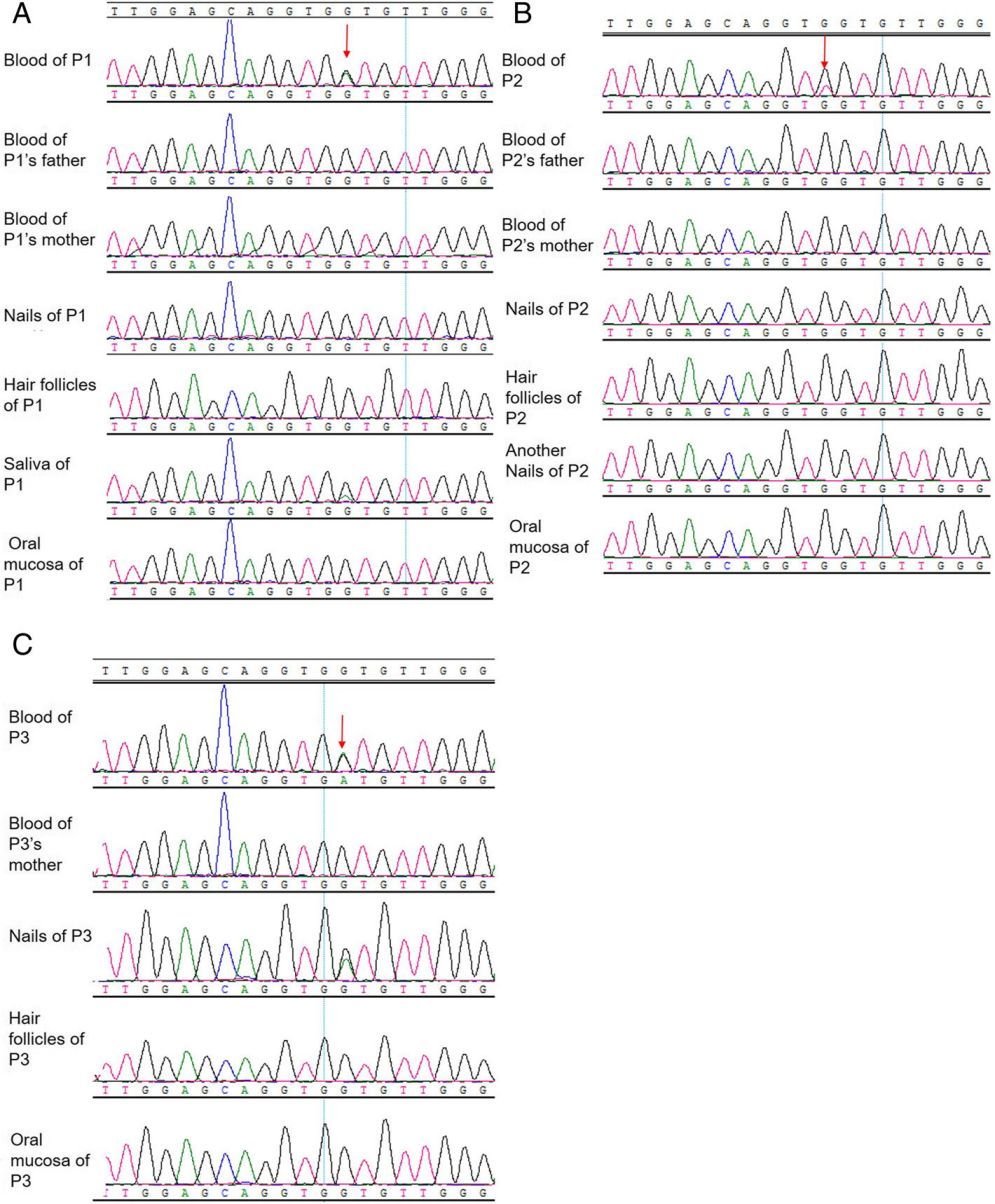
Direct sequencing of NRAS genes in different tissues from patients. Blood samples, oral mucosa cells, hair follicles, and nails were collected from each patient as well as blood samples from their parents, and sequenced. **a** Mutated NRAS gene (c.38G > A,p.G13D) was found only in the blood sample in Patient 1 as well as in saliva, which had a lower frequency of mutated allele. No mutation was found in either other tissues of Patient 1 or in blood of his parents. **b** Mutation of NRAS (c.37G > T, p.G13C) was only detected in blood of Patient 2, compared to the results of his other tissues and blood from his parents. **c** Mutated NRAS gene (c.38G > A,p.G13D) was found in the blood and nails of Patient 3 while NRAS genes in her hair follicles, oral mucosa as well as in her mother’s blood are all normal

The clinical presentations and laboratory examination data for all 3 patients were summarized in Table 1. And the genetic results were showed in Fig. 1.

**Table 1:** Clinical presentations and Laboratory data at diagnosis in those patients

	Patient 1	Patient 2	Patient 3	Normal
Gender	Male	Male	Female	
Age of onset (year)	3	1	4	
Clinical presentation& Physical examination				
Family history	–	–	–	
Fever	+	+	–	
Malar rash	+	–	+	
Lymphadenopathy	+	+	–	
Hepatomegaly	+	+	+	
Splenomegaly	–	+	+	
Arthritis	+	+	+	
Pericardial effusion	+	–	–	
Proteinuria	–	–	+	
Peripheral blood examination				
WBC(×10^9^/L)	5.16–11.73	4.01–8.38	3.45–10.44	3.5–9.5
Neutrophil (%)	37.5–64.3	53.6–76.3	40.6–82.1	50.0–75.0
Lymphocyte (%)	24–45.7	17.8–38.6	10.7–29.9	20.0–40.0
CD3+ cells (%, cells/ml)	79.9, 2335	86.6, 1438	NA	60.05–74.08, 1424–2664
CD3 + CD4+ cells	35, 1023	40.9, 679	NA	26.17–40.76, 686–1358
CD3 + CD8+ cells	40.8, 1193	36.4, 604	NA	19.68–34.06, 518–1125
CD20+ B cells	13.8, 403	6.1, 101	NA	10.21–20.12, 280–623
Double negative T cells (%) (TCRαβ+CD3 + CD4-CD8-)	4.9	7.2	NA	0.18–2.81
Monocyte (%)	8.8–18.3	2.4–10.9	6.9–28.6	3.0–8.0
Monocyte	0.69–1.79	0.2–0.83	0.45–2.4	0.12–0.80
Hemoglobin (g/dL)	5.7–10.4	12.1–15.8	10.5–15.7	12.0–16.0
RET%	4.07	1	1.46	0.8–2.0
Platelet (×10^9^/L)	24–366	28–171	14–189	100–350
CRP (mg/dL)	< 0.1	0.3	< 0.1	0–0.8
ESR (mm/h)	51	19	31	0–15 for boys 0–20 for girls
Double negative T cells (%) (TCRαβ+CD3 + CD4-CD8-)	4.9	7.2	NA	
IgG (mg/dL)	2331	1629	2158	500–1200
IgM (mg/dL)	625	201	56	40–230
IgA (mg/dL)	312	133	743	70–400
C3 (g/L)	< 0.406	0.251	↓	0.73–1.46
C4 (g/L)	< 0.046	0.010	↓	0.1–0.4
ANA	H1:1280	H1:640	S1:1280	< 1:40
Anti-dsDNA-ELISA (U/mL)	664	770	140	< 100
Anti-dsDNA-IF	+ 1:320	+ 1:40	–	< 1:5
Anti-Sm	–	–	+(1:4)	–
ACL (U/mL)	17	–	–	< 12
β2GP1-IgA (U/mL)	54	–	–	< 20
Coombs	+	+	+	–
EB IgG/VCA	+ 5.62	+ 7.93	NA	< 0.80
EB IgM/VCA	+ 1.31	–	NA	< 0.80
EBV-DNA	+	–	NA	< 500
CMV-IgM	+ 3.18	+ 1.86	+ 1.31	< 0.9
CMV-IgG	NA	+ 3.64	+ 4.79	< 0.9
CMV-PP65	+	+	–	–

Patient 1 was a 4-year-old boy hospitalized due to lymphadenopathy and anemia for 9 months and thrombocytopenia recently in August 2016. When he was 3 years and 10 months old, his patients occasionally found enlarged lymph nodes in left neck with tenderness, accompanied with intermittent fever. Laboratory data revealed anemia with coombs test (+), and elevated CRP. Bone marrow and lymph node biopsy were both negative. He received antibiotics and oral prednisone treatment, which achieved remission of symptoms. However, those symptoms recurrently occurred once the dose of prednisone decreased. In March 2016, after a respiratory infection, He gradually developed arthralgia, lymphadenopathy, anemia and red maculopapulae spread from legs to whole body, which remained pigment deposition in most lesions and facial erythema after anti-allergy treatment. Then, in July, the patient developed intermittent high fever. Blood routine examination found anemia (HGB 56 g/L), and progressive thrombocytopenia (lowest Plt 24 × 10^9^/L). Laboratory examinations for immune system suggested positive ANA (ANA>H1:1000), anti-nucleosomes antibodies, anticardiolipin antibody (ACL), and weak positive anti-ribosome protein P, as well as decreased complements (C3 < 0.169 g/L, C4 < 0.066 g/L). Besides, echocardiography observed enlarged left atrial and left ventricle, widened left coronary artery and pericardial effusion. He was diagnosed as SLE in local hospital for facial maculopapulae, arthritis, pericardial effusion, hemolytic anemia and severe thrombocytopenia, accompanying with high titers of antinuclear antibody (ANA), anticardiolipin antibody (ACL), as well as decreased complements, according to the Systemic Lupus International Collaborating Clinics (SLICC) criteria [5]. He was also diagnosed as EBV-infection because of high copies of EBV-DNA. He received IVIG for 3 days and high dose of corticoids accompanied with anti-virus treatment. The patient was admitted to PUMCH for further investigation. His parents denied any similar symptoms. Physical examinations found facial erythema, cervical lymphadenopathy in both sides, and hepatomegaly. Results of laboratory examinations were quite similar to previous results, except that CMV-IgM as well as CMVPP65 was positive this time. Considering of his age and gender, although he met the classification criteria of SLE, considering of his age and gender, it should be careful to make the diagnosis. Thus, we made a further investigation. We found obvious monocytosis in routine blood examination, elevated IgG level, and increased CD3 + TCRαβ+CD4-CD8- (αβ-DNT) cells by peripheral blood immunophenotyping. Combining with his recurrent virus-infection history, there is a high possibility of PIDs, especially for lymphoproliferative diseases. Therefore, we performed genetic sequencing using our PIDs panel and a neuroblastoma RAS viral oncogene homologue (NRAS) mutation of c.38G > A,p.G13D was detected in the blood. Besides, the same set of mutations was detected in his buccal mucosa of patient 1, while the frequency was much lower (Fig. 1a). However, no mutation was found in other tissues, either from him or his parents, which suggested he carried somatic mutations instead of germline mutations. He was treated with a tapering dose of prednisone and the dose of prednisone was 7.5 mg qd at his last visiting, without thrombocytopenia anymore.

Patient 2 was 7 years old when he came to our hospital for splenomegaly and thrombocytopenia for 6 years. When he was 11 months old, he was hospitalized because of recurrent fever (Tmax 38.9 °C), cough, and dyspnea. At that time, splenomegaly and thrombocytopenia were found. Since then, he had suffered from 5 to 6 episodes of cough and dyspnea every year, sometimes accompanied with arthralgia. Half a month before he was admitted to PUMCH, he suffered a new episode of fever and cough, and was hospitalized in local hospital because of severe thrombocytopenia (PLT 28 × 10^9^/L). Laboratory examinations found ANA+++ and positive ANA. He was diagnosed as SLE in local hospital and treated with methylprednisolone 30 mg for 2 days, accompanying platelet transfusion once. And the patient was admitted to our hospital then. No positive family history was observed. Physical examination found cervical lymphadenopathy and hepatosplenomegaly. Besides, dry rale as well as wet rale and wheezing rale can be heard all around both sides of lung. Routine blood examination showed slight thrombocytopenia (PLT 97 × 10^9^/L). Immune system laboratory test showed ANA(+)H1:1280, and anti-dsDNA antibody (+) (DNA-ELISA 770 IU/ml, and DNA-IF (+)1:40), rRNP (+), decreased complement, and positive results of Coombs test. And joint ultra sound examination supported the existence of arthritis. Abdominal ultrasound examination and CT found hepatosplenomegaly. And ECHO found enlarged left atrial. He met 5 of SLICC criteria: thrombocytopenia, arthritis, ANA (+), dsDNA (+) as well as decreased complement. In terms of infection, this patient had CMV-IgM, and CMVPP65 (+). Thoracic HRCT indicated bronchiectasis in upper and middle lobes in right lung as well as in lower lobes in both sides. Multiple ground glass shadow and rope stripe shadow were also seen in both sides of lung. His recurrent infectious history since the first year of life strongly supported the possibility of PIDs. We performed Gene trapping high-throughput sequencing using blood samples from him and his parents, and a mutation of NRAS, c.37G > T, p.G13C was found in his blood, while no mutation was found in his parents’ blood, either in other tissues of him by sequent Sanger sequencing. Other evidences supporting the diagnosis of RALD included monocytosis in peripheral blood, and increased serum IgG. Peripheral blood immunophenotyping showed that αβ-DNT cells proportion was highly elevated to 7.2% in peripheral blood. He was treated with prednisone and leflunomide (LEF) to help reduce the dose of prednisone. During his last visit, most symptoms were relieved except hepatosplenomegaly with the treatment of prednisone 5 mg qod and LEF 10 mg qod.

Patient 3 came to PUMCH at the age of 6 because of thrombocytopenia. The onset symptoms of her disease were purpura in legs along with thrombocytopenia before she was 4 years old. She was diagnosed as immune thrombocytopenic purpura (ITP) at that time and received corticoids treatment, which was effective. Later, she gradually developed facial malar rash with photosensitivity, arthralgia, and edema in lower limbs and eyelids. Considering the above symptoms and following positive laboratory data: proteinuria (24 h urine protein was 3.3 g), thrombocytopenia, positive Coombs test, positive ANA, ds-DNA as well as anti-Sm antibody, she was diagnosed as SLE at local hospital three months before she was admitted PUMCH. She was treated with IVIG 7.5 g for 5 days, methylprednisolone 0.6 g i.v. for 3 days followed by prednisone 35 mg qd, and CTX 0.5 g per month twice. Her past medical history was negative except that she was infected with mumps at the age of 5. Physical examination suggested slight malnutrition, and hepatosplenomegaly, which was further proved by ultrasound. Routine blood examination showed normal results and 24hUP was 1.27 g. The CMV-IgM was positive, indicating a possible virus infection. Other examinations in autoimmune diseases got similar results to that of local hospital. Initially, we agreed with the diagnosis of SLE and continued the therapy of prednisone and immunosuppressant (CTX for 5 months and followed by mycophenolate mofetil (MMF), accompanied with hydroxychloroquine (HCQ)). About 9 months later, all of her symptoms were relieved. However, persistent hepatosplenomegaly and increased ESR were observed, which motivated us to perform the genetic sequencing for PIDs. A NRAS mutation of c.38G > A,p.G13D was identified in her blood. After further Sanger sequencing identification, this mutation was only found in hematopoietic cells and a minor portion of cells in her nails. No mutations were detected in other tissues of her, or her parents. We also found monocytosis in routine blood examinations and elevated IgG level, which favored the diagnosis of RALD. The dose of prednisone was reduced to 5 mg qd at her last visit.

## Discussion

NRAS is a member of RAS family which plays an important role in several intracellular signaling pathways like proliferation and apoptosis [6]. It is well known that in melanoma, pancreatic cancer, lung cancer, bladder cancer and acute myeloid leukemia, NRAS mutations are commonly found [7]. Apart from causing neoplasm, germline mutations in RAS genes, such as G60E in NRAS, can result in RASopathies, which include Noonan syndrome and Noonan-related syndromes [8, 9]. The fundamental clinical features of RASopathies consist of growth retardation, several special facial features, congenital heart diseases, skeletal malformations and increased risk for neoplasms [10]. Previous studies highlighted a relation between RASopathies and autoimmunity as in 52% of 42 patients diagnosed as RASopathies, autoimmune antibodies were detected [11]. However, we didn’t find the typical clinical manifestations as well as germline mutations of NRAS in our patients, and therefore, those patients were not considered as RASopathies. However, patients carrying somatic NRAS mutations mainly in hematopoietic lineage cells were reported to be highly likely to develop RALD, when the mutations cause amino acid substitutions in codons 12 or 13 [12]. So far, only a small number of patients with RALD were reported [4, 12–17].

RALD was initially classified into the type IV of autoimmune lymphoproliferative syndrome (ALPS) caused by KRAS/NRAS mutation [4]. Recent studies suggested that patients with RALD may have elevated or normal αβ-DNT cells level, while in ALPS patients, increased αβ-DNT cells is one of the most specific findings [4, 13, 18]. Thus, in 2009, a new nomenclature of RALD was used to describe this disease [19].

SLE mostly affects women of reproductive age, only about 10 to 20% of patients of SLE are diagnosed since childhood, which is called early-onset SLE [20–22]. Early-onset SLE patients usually manifest with typical lupus rashes (77%), constitutional features (fever, myalgia, fatigue, weakness, weight loss) (55%), renal diseases (44.4%), and arthritis/arthralgia (38.8%) [23]. In terms of laboratory examinations, positive ANA titers and anti-dsDNA are found in almost all patients (> 90%), while decreased complements are also common (about 70%) [24].

In terms of these three RALD patients, they were presented with rashes (3/3, 100%), arthritis (3/3, 100%), severe thrombocytopenia (3/3, 100%). Besides, patient 1 developed pericardial effusion while patient 3 had nephritis. In terms of laboratory examinations, Coombs test (+), decreased complement, high titers of ANA, as well as anti-dsDNA, were found in all patients (100%), while ACL and β2GP1 were positive in patient 1 and anti-Sm were found in patient 3. Those findings led to the diagnosis of SLE in local hospitals, according to SLICC criteria.

However, we noticed that there were several uncommon manifestations in early-onset SLE. First, those patients were less than 5 years old at the onset of their diseases, while the median age at SLE onset was about 12 years (mean, 11.5 ± 2.5), and 85% SLE developed after 8 years old [24]. Second, consistent with the fact that female is more likely to develop SLE compared to male, the girl-to-boy ratio is 4.5:1 among early-onset SLE patients. However, patient 1 and 2 in our case series are both boys, and the girl-to-boy ratio is 1:2. Third, while hepatosplenomegaly and lymphadenopathy were reported to show up in only about 22 and 33% of early-onset SLE patients, respectively [23], those patients had hepatomegaly (3/3, 100%) with/without splenomegaly (2/3, 67%) or lymphadenopathy (2/3, 67%), and the symptoms of hepatomegaly as well as splenomegaly did not alter after the patients received treatment as SLE. Besides, all patients suffered from recurrent infections, which reminded us of the possibility of PIDs. Moreover, monocytosis was observed in all patients, which was a characteristic feature of RALD [12].

Combining all of those unusual clinical presentations, we decided to perform a Gene trapping high-throughput sequencing of PIDs screening to verify the possibility of PID. Fortunately, we did find the NRAS mutations in their hematopoietic cells and further investigations proved that all of them carried somatic NRAS mutations, which supported the final diagnosis of RALD.

Thus, by summarizing the clinical presentations of our RALD patients and reviewing literature about RALD as well as early-onset SLE, we propose that suspected SLE patients should be carefully considered for the possibility of RALD, if they meet the following requirements:

1. The disease onsets at an early age, like < 5 years old, especially for male patients.
2. Severe hematologic changes are observed, including thrombocytopenia, and anemia, accompanied with Coombs test (+).
3. Physical examinations or image examinations such as BUS or CT suggest obvious and persistent hepatosplenomegaly and/or lymphadenopathy.
4. Monocytosis.
5. Any clues that suggest the possibility of PIDs such as recurrent infection history.

Elevated serum IgG is usually found in RALD patients, although it can be seen in some SLE patients. Peripheral blood immunophenotyping might provide more evidence for RALD if elevated αβ-DNT cells is found, which is not usually mentioned in SLE but often reported in RALD [15, 16]. We would need a genetic examination for final diagnosis.

As RALD is often caused by NRAS or KRAS somatic mutations in hematopoietic cells, detecting NRAS or KRAS mutations by genetic detection of blood samples should be the first step. If a NRAS or KRAS mutation is found, it is necessary to take the second step to test other tissues like hair follicles, saliva, nails, and oral mucosa cells, in order to confirm whether the mutation is somatic.

## Conclusions

We reported three Chinese cases with RALD caused by somatic NRAS mutations. By concluding the major characteristics of the patients, we pointed out that many RALD patients might be misdiagnosed, and we also came up with an assumption to screen RALD patients out of early-onset SLE patients.

## Data Availability

Please contact authors for data requests.

## Abbreviations

ACL: Anticardiolipin antibody
ALPS: Autoimmune lymphoproliferative syndrome
ANA: Antinuclear antibody
BUS: B-Mode ultrasonography
PIDs: Primary immunodeficiency diseases
PUMCH: Peking Union Medical College Hospital
RALD: RAS-associated Autoimmune Leukoproliferative disease
SLE: Systemic lupus erythematosus
SLICC: Systemic Lupus International Collaborating Clinics
αβ-DNT: CD3^+^TCRαβ^+^CD4^−^CD8^−^
β2GP1: Anti-β2-glycoprotein I
